# On the Impact of Left Upper Lobectomy on the Left Atrial Hemodynamics

**DOI:** 10.3389/fphys.2022.830436

**Published:** 2022-02-24

**Authors:** Tomohiro Otani, Takuya Yoshida, Wentao Yi, Shunsuke Endo, Shigeo Wada

**Affiliations:** ^1^Graduate School of Engineering Science, Osaka University, Osaka, Japan; ^2^Jichi Medical University Saitama Medical Center, Saitama, Japan

**Keywords:** left atrium, hemodynamics, left upper lobectomy, pulmonary vein, computational fluid dynamics, Lagrange coherent structure

## Abstract

The left atrium (LA) functions to transport oxygenated blood from the pulmonary veins (PVs) to the left ventricle (LV). LA hemodynamics has received much attention because thrombosis in the LA in pathological states, such as atrial fibrillation, is a major factor leading to thromboembolic stroke. In the last 5 years, multiple cohort studies have revealed that left upper lobectomy (LUL) with PV resection risks thrombus formation in the PV stump even in the normal LA without a history of cardiac disease; the causal mechanism is, however, an open question. The present study investigated the potential effect of an LUL on LA hemodynamics associated with thrombus formation through computational simulation using four-dimensional computed tomography (4D-CT) images. Time series of patient-specific LA geometries before and after LUL were extracted from the 4D-CT images and these motions were estimated through non-rigid registration. Adopting the LA geometries and prescribed moving wall boundary conditions, the LA blood flow was determined using a Cartesian-grid computational fluid dynamics solver. The obtained results show that the LUL resulted in blood flow impingement from the left and right PV inflows into the LA upper region throughout most of the cardiac cycle. This characteristic alteration of the LA hemodynamics generated fine-scale vortices with viscous energy dissipations, enhancing the flow stasis associated with thrombus formation in the PV stump. These findings show that an LUL affects the hemodynamics not only in the PV stump but also throughout the LA region. They also highlight the importance of computational analysis of LA hemodynamics in understanding the underlying mechanism of LUL-induced thrombus formation.

## 1. Introduction

The left atrium (LA) is the left upper heart chamber and transports oxygenated blood from the pulmonary veins (PVs) to the left ventricle (LV) through the mitral valve (MV). LA pathological states, such as atrial fibrillation, are risk markers of thrombus formation in the LA, which is associated with thromboembolic stroke (Wolf et al., 1991). The LA hemodynamics in atrial fibrillation have recently attracted attention, and several studies have conducted computational simulations of LA blood flow analysis aimed at clarifying causal relationships between LA hemodynamics and thrombosis (Otani et al., 2016; Bosi et al., 2018; Dillon-Murphy et al., 2018; Masci et al., 2019, 2020; Dueñas-Pamplona et al., 2021). These studies have advanced computational approaches to modeling LA hemodynamics by considering anatomical LA geometries, physiological motions, and boundary conditions (Lantz et al., 2018, 2019; Otani et al., 2019).

However, even in cases with a normal LA and no prior history of atrial fibrillation, recent cohort studies have clarified that a lobectomy during thoracic surgery has a risk of LA thrombosis leading to thromboembolic stroke as a short-term complication (Yamamoto et al., 2016; Hattori et al., 2019; Riddersholm et al., 2019; Xie et al., 2019). A lobectomy is conducted for patients with lung cancer and requires an associated PV resection from the LA. Thrombus formation in the PV stump (with the remaining PV resected) after a lobectomy was reported for 3.6% of 193 patients (Ohtaka et al., 2013) and reported to lead to a rare but potentially lethal cerebral infarction for approximately 1% of patients 30 days after lobectomy (Yamamoto et al., 2016; Riddersholm et al., 2019). In particular, left upper lobectomy (LUL) with left superior PV (LSPV) resection has a relatively high risk of thrombus formation (Ohtaka et al., 2013; Riddersholm et al., 2019; Xie et al., 2019). The cause of thrombosis may be mechanical (Mumoli and Cei, 2012), and thus hemodynamic factors, such as flow stasis and disturbances primarily in the LSPV stump, may be associated with thrombus formation (Ohtaka et al., 2014a,b).

Nevertheless, alterations of LA hemodynamics following lobectomies are still poorly understood, and existing studies of LA hemodynamics after an LUL are limited to two magnetic resonance imaging (MRI) measurements made by the same research group (Matsumoto et al., 2020; Nakaza et al., 2021), to the best of our knowledge. These studies reported decreases in the flow velocity in and around the PV stump (Matsumoto et al., 2020) and alterations of the flow pathline features in the LA (Nakaza et al., 2021), but the spatiotemporal resolution was too low to capture the detailed LA hemodynamics, especially in the PV stump. To address these shortcomings, computational studies of LA blood flow can be used to deepen our understanding of possible LA hemodynamics after a lobectomy.

In this study, we used computational simulations to investigate a possible alteration of the LA hemodynamics by an LUL. Using our previously developed electrocardiogram-gated four-dimensional computed tomography (4D-CT) framework (Otani et al., 2016, 2019), a personalized blood flow simulation in the LA was conducted for a lung cancer patient before and after an LUL. This study is the first to investigate the effect of an LUL on LA hemodynamics and shows they are altered in the PV stump and throughout the LA region.

## 2. Materials and Methods

### 2.1. CT Image Acquisition and Processing

This study used electrocardiogram-gated 4D-CT images of a patient (male, 66 years of age) with lung cancer and no prior history of cardiac disease who received an LUL at the Department of Thoracic and Cardiovascular Surgery at Jichi Medical University Hospital. Thrombus formation in the PV stump was not found for this patient after the LUL. The study was approved by the Institutional Review Board of Jichi Medical University. The patient gave oral and written informed consent to participate in this study.

The CT images were acquired for a normal sinus rhythm using a 128-slice multi-detector CT scanner (SOMATOM Definition Flash, SIEMENS, Inc., Berlin, Germany) and reconstructed for 20 phases during the cardiac cycle from the electrocardiographic ventricular end-diastole. The in-plane pixel dimensions were 0.39 × 0.39 mm^2^ and the through-plane slice thicknesses and increments were both 1.0 mm. CT images were acquired 4 days before and 16 days after the LUL. The cycle length, *T*, was 0.92 s (65 bpm) and 0.82 s (73 bpm) before and after the lobectomy, respectively.

**Figure 1:**
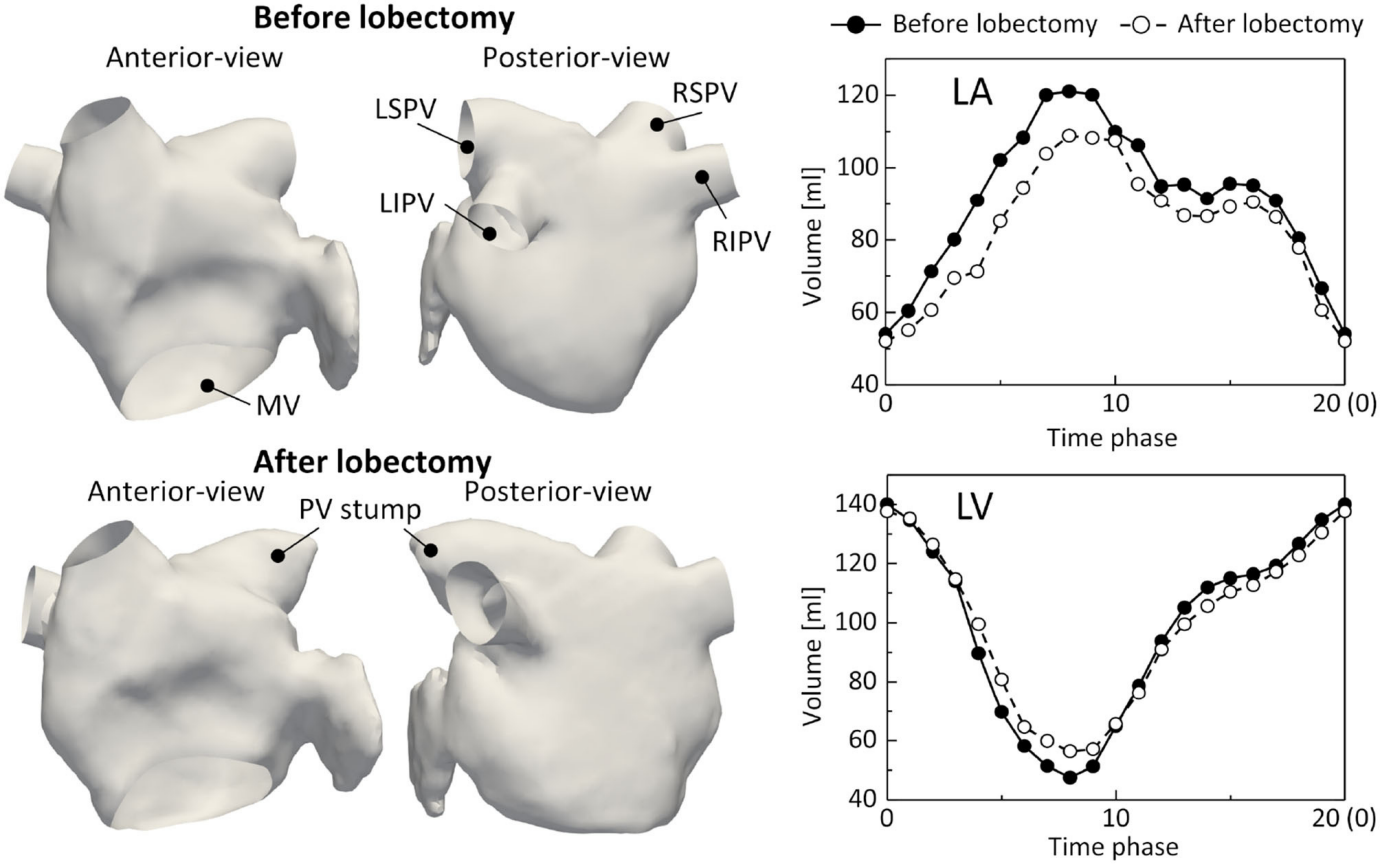
Left atrium (LA) surfaces at phase 0 (left) and time courses of the LA and left ventricle (LV) volumes (right) before and after lobectomy, extracted from 4D-CT images. LSPV, left-superior pulmonary vein; LIPV, left inferior pulmonary vein; RSPV, right superior pulmonary vein; RIPV, right-inferior pulmonary vein, and MV, mitral valve.

The LA and LV regions were segmented from the 4D-CT images at all phases using the Mimics cardiovascular segmentation tool (Version 23; Materialize, Inc., Yokohama, Japan). The LA surfaces were reconstructed using a set of linear triangular elements (base-mesh resolution: 1.5 mm), where the distal part of the PVs beyond the first bifurcation and the MV were removed using 3-Matic (Version 15; Materialize, Inc., Yokohama, Japan). In addition, the LV volume was calculated at all phases to compute time courses of blood flux from the LA to the LV. Figure 1 shows the LA surfaces and time courses of the LA and LV volumes before and after the LUL. The LSPV was resected by the LUL and the LSPV stump remained. The LA and LV volumes and their time courses were similar before and after the lobectomy. Notably, the connecting direction of the left inferior PV (LIPV) to the LA was different before and after the LUL, which is known to result from remodeling of the remaining lung (Nakaza et al., 2021).

### 2.2. Left Atrial Surface Processing

**Figure 2:**
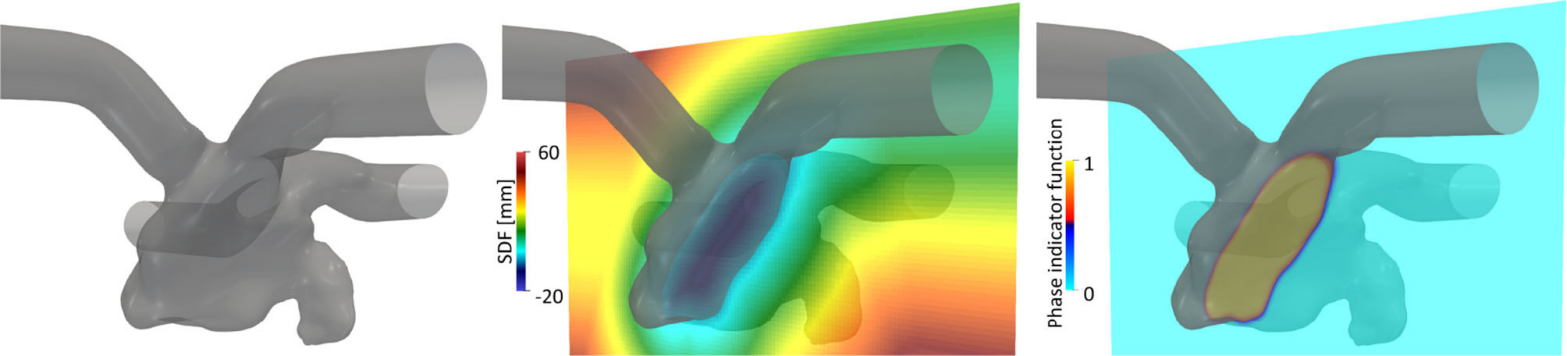
Left atrial surface (left) and spatial distributions of the signed distance function (SDF), ϕ (center), and phase indicator function, κ, computed using the SDF (right) in a representative cross-sectional plane.

The displacement of the LA surface throughout the cardiac cycle was estimated through non-rigid point set registration using a coherent point drift algorithm (Myronenko and Song, 2010) adjusted for the estimation of LA motion, as described in our previous study (Otani et al., 2019). The estimated displacements of the LA surface and LV volume were temporally interpolated using the inverse Fourier expansion within fifth-order modes. Additionally, velocity vectors were obtained by differentiating the displacement vectors with respect to time. For subsequent computational fluid dynamics simulation, cylindrical tubes were attached to the PV and MV sections to weaken the effects of setting artificial boundary conditions on the LA hemodynamics [Figure 2 (left)]. Here, the displacement field in cylindrical tubes was smoothed by solving the steady-state Laplace equation with the LA displacement field estimated above using the finite element method, as described previously (Otani et al., 2016).

### 2.3. Computational Simulation

The computational fluid dynamics simulation of the LA hemodynamics was conducted by a Cartesian grid solver using a boundary data immersion (BDI) method (Weymouth and Yue, 2011) described in our previous study (Otani et al., 2018). The BDI method solves the meta equation, which is derived through phase mixing of the governing equations for the fluid and solid with the prescribed velocity field, using a phase indicator function κ on a Cartesian grid. To apply the BDI method to LA blood flow simulation, we represented the LA surface implicitly using a signed distance function (SDF) ϕ(**x**) at each position vector of the Cartesian grid **x**. The SDF is given by


(1) $$\begin{array}{l} \phi \left(\text{x}\right)=\{\begin{array}{cc} \text{min}|\text{x}-\hat{\text{x}}| & \text{in }\Omega_{\text{f}}\\ 0 & \text{in }\Gamma ,\;\hat{\text{x}}\in \Gamma \\ -\text{min}|\text{x}-\hat{\text{x}}| & \text{in }\Omega_{\text{s}}, \end{array} \end{array}$$

where Ω_f_ and Ω_s_ are the fluid and solid domains, respectively, and Γ is their interface [Figure 2 (center)]. Inter-sectional points between LA surface triangles and the Cartesian grid were detected in a manner similar to that adopted by Mittal et al. (2008), and the SDF was constructed using the narrowband fast marching method (Sethian, 1999) with MPI parallelization proposed by Yang and Stern (2017). In this study, the phase indicator function κ(ϕ) was expressed as a smooth interface using the SDF (Sethian and Smereka, 2003):


(2) $$\begin{array}{l} \kappa \left(\phi \right)=\{\begin{array}{cc} 0 & \text{if }\phi <-\varepsilon \\ \frac{1}{2}\left[1+\frac{\phi }{\varepsilon }+\frac{1}{\pi }\sin\left(\frac{\pi \phi }{\varepsilon }\right)\right] & \text{if }|\phi |\leq \varepsilon \\ 1 & \text{if }\phi >\varepsilon , \end{array} \end{array}$$

with the interface thickness, ε, set to the cell size [Figure 2 (right)]. In addition, the LA surface velocity field estimated from Section 2.2 was extrapolated in Ω_*s*_ and Γ on a Cartesian grid and denoted **u**_LA_(**x**, *t*). We assumed that **u**_LA_ around the LA surface was normal to the wall boundary (= ∇ϕ) and extrapolated it on the Cartesian grid by solving the advection equation with a given LA surface velocity, following (Peng et al., 1999).

We treated the blood as an incompressible Newtonian fluid because a non-Newtonian effect is secondary in evaluating LA blood flow (Ku, 1997). The governing equations in the fluid domain, Ω_f_, are


(3) $$\begin{array}{l} \rho \left(\partial_{t}\text{u}+\text{u}\cdot \nabla \text{u}\right)=-\nabla p+\eta \nabla^{2}\text{u}, \end{array}$$

(4) $$\begin{array}{l} \nabla \cdot \text{u}=0, \end{array}$$

where **u** is the velocity vector, *p* is the pressure, ρ is the density, and η is the dynamic viscosity. We solved Equations (3) and (4) using the BDI method (Weymouth and Yue, 2011) in a finite difference manner using a conventional staggered grid. In the temporal discretization, the convection and diffusion terms were treated explicitly, whereas the pressure term was treated implicitly using fractional step method. In the spatial discretization, the convection term was treated using the fifth-order weighted essentially non-oscillatory (WENO) scheme, and a second-order central difference scheme was used for all other spatial derivatives. The pressure Poisson equation was solved using the biconjugate gradient stabilized (BiCGSTAB) method preconditioned by the geometric The density and dynamic viscosity were set at ρ = 1.05 × 10^3^ kg/m^3^ and η = 3.5 × 10^−3^ Pa·s. For the MV boundary, we considered the MV to be closed during ventricular systole (assuming a wall boundary) and open during ventricular diastole, and the time required for MV opening and closing to be negligible. When the MV was open, we set the time-varying flow rate with a uniform velocity profile determined by the LV volume changes [Figure 1 (bottom-right)]. No specific model was considered for the geometry of the MV leaflets (Vedula et al., 2015). At the PV cross-sections, we set a uniform pressure *P*_PV_ for each PV using the terminal vessel resistance model, simply expressed by *P*_PV_ = *P*_0_ + *KQ*_PV_, where *P*_0_ is the baseline, *K* is the terminal resistance, and *Q*_PV_ is the flux through the PV cross-section (positive for inflow and negative for outflow). An equal flow rate through all PVs most closely resembles *in vivo* measurements according to Lantz et al. (2019), and we thus set *P*_0_ = 0 Pa and *K* = 100 MPa s/m^3^, which provide a nearly equal flow rate through all PVs before the lobectomy. In preliminary simulations, we confirmed that flow rate differences were within 5 mL/s in each PV before the LUL. On the wall boundaries, the no-slip condition was implicitly adopted using the BDI formulation.

Each CFD computation was iteratively conducted for three cardiac cycles. The pressure Poisson equation was judged to have converged when the residual was less than 10^−4^ in a time step. The time increment was chosen to satisfy a Courant–Friedrichs–Lewy (CFL) number less than 0.25. The number of cells was set at 512 × 512 × 512 with a resolution of 0.3 mm in each dimension. The grid size dependence was examined in a preliminary simulation, as described in the Appendix. We performed computational simulations of the LA blood flow before and after the LUL under hybrid parallelization with 611 (before) and 627 (after) MPI nodes and 12 OpenMP cores using the Fugaku supercomputer (RIKEN, Kobe, Japan). Each CFD simulation of three cardiac cycles took approximately 8 h. The next section presents computational results for the third cardiac cycle, unless otherwise noted.

## 3. Results

**Figure 3:**
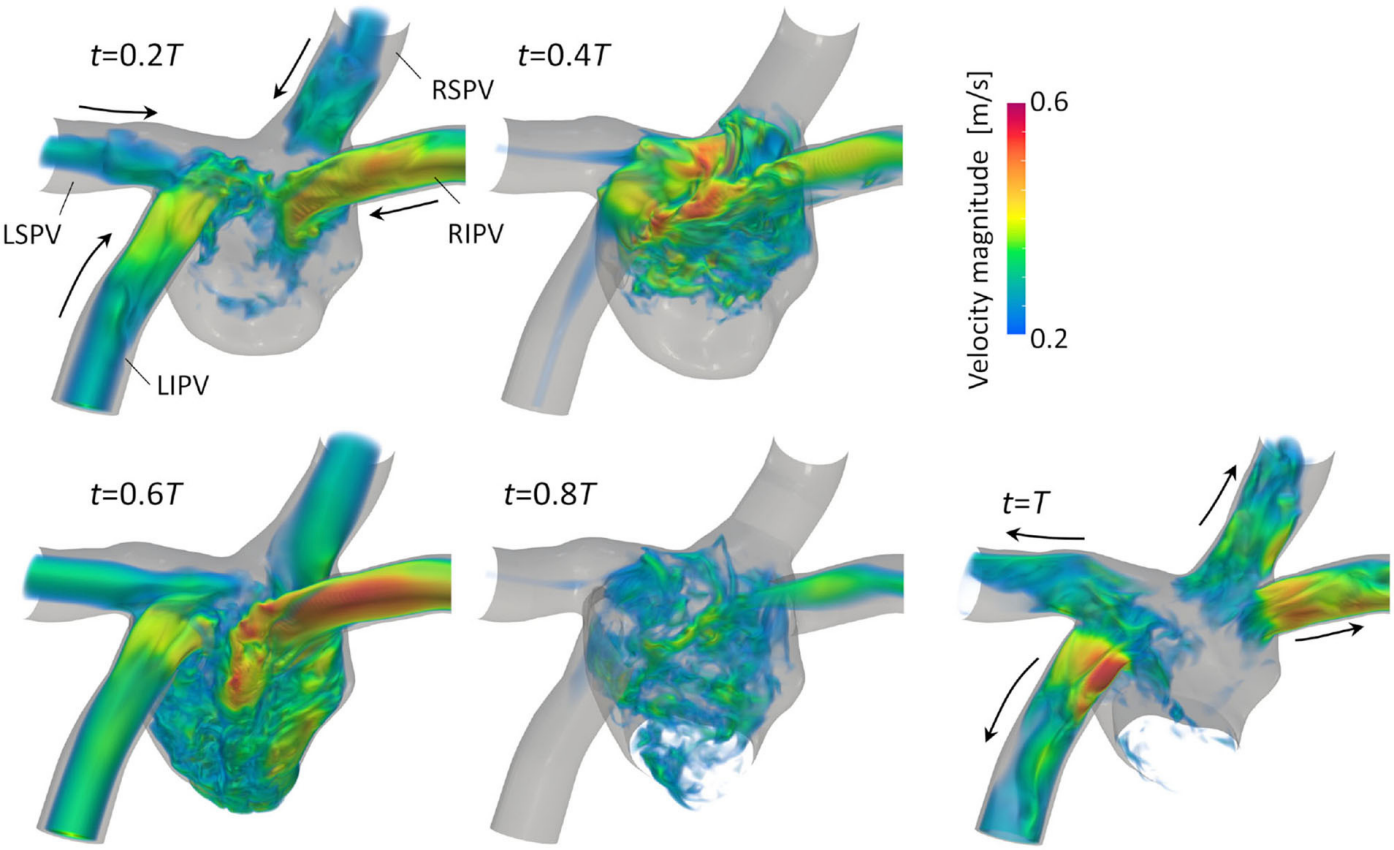
Posterior views of the velocity distribution with volume rendering at *t* = 0.2*T*, 0.4*T*, 0.6*T*, 0.8*T*, and *T* before upper-left lobectomies (*T*: cardiac cycle from the start of systole). Refer to Supplementary Video 1 to see the velocity transition for a whole cardiac cycle.

**Figure 4:**
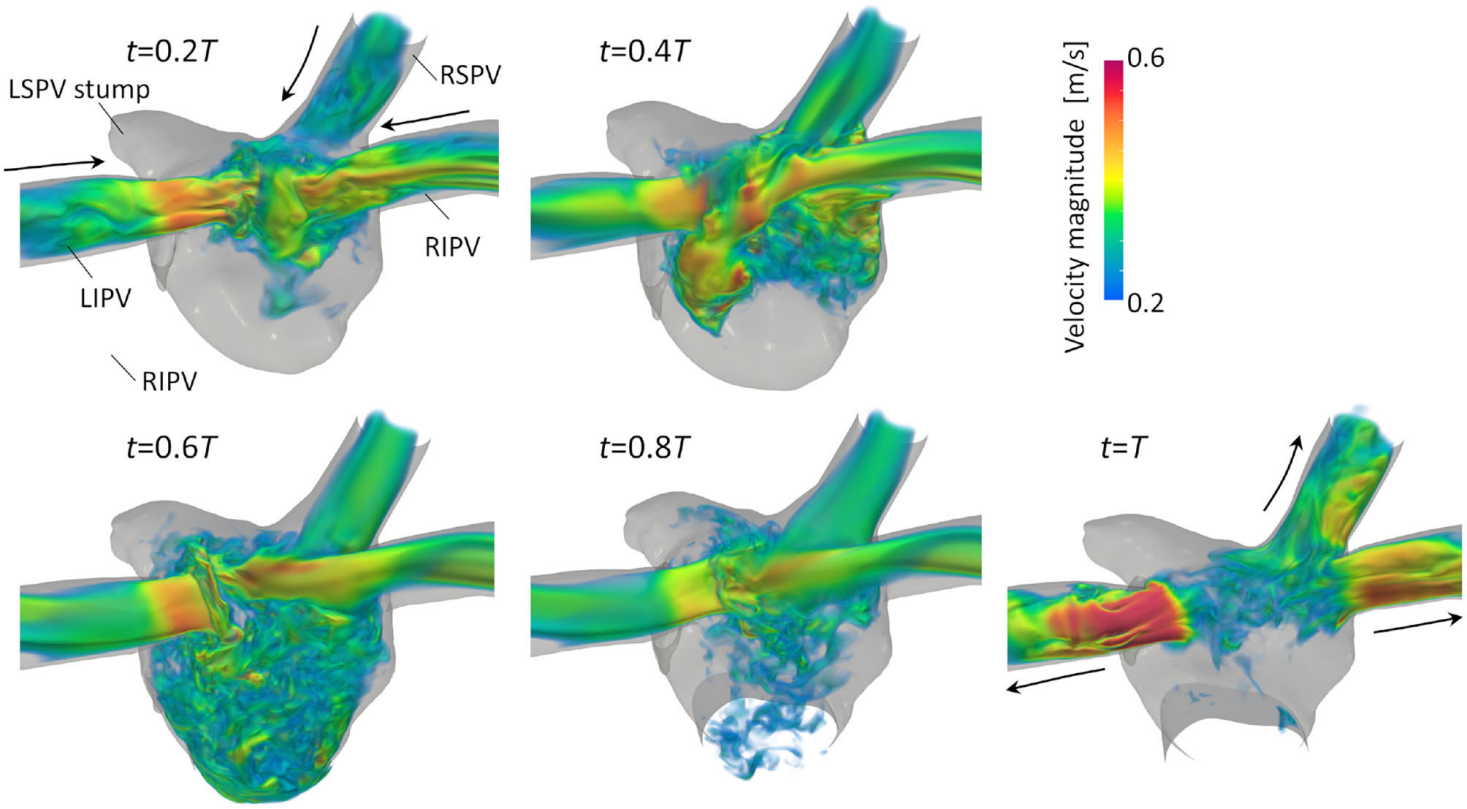
Posterior views of the velocity distribution with volume rendering at *t* = 0.2*T*, 0.4*T*, 0.6*T*, 0.8*T*, and *T* after LULs (*T*: cardiac cycle from the start of systole). Refer to Supplementary Video 1 to see the velocity transition for a whole cardiac cycle.

Figure 3 illustrates the distribution of the LA blood flow velocity before the LUL with volume rendering to intuitively show the global LA hemodynamics in a cardiac cycle; refer to Supplementary Video 1 to see the distribution for a whole cardiac cycle. From the early systole, the blood inflow from each PV increased the LA volume, and the velocity magnitude increased mainly in the LA upper region (*t* = 0.2*T*, 0.4*T*). At the beginning of the LV diastole, LA blood flow exited to the LV through the mitral annulus. The blood flow velocity increased throughout the LA at early-diastole (*t* = 0.6*T*) and then decreased at mid-diastole (*t* = 0.8*T*). Finally, there was reverse flow in the PVs owing to the atrial kick at end-diastole (*t* = *T*). Meanwhile, the LA hemodynamics after the LUL had tendencies different from those before the LUL [Figure 4]. Flow impingement from the right PVs and remaining LIPV in the upper region of the LA appeared throughout the cardiac cycle except the end-diastole.

**Figure 5:**
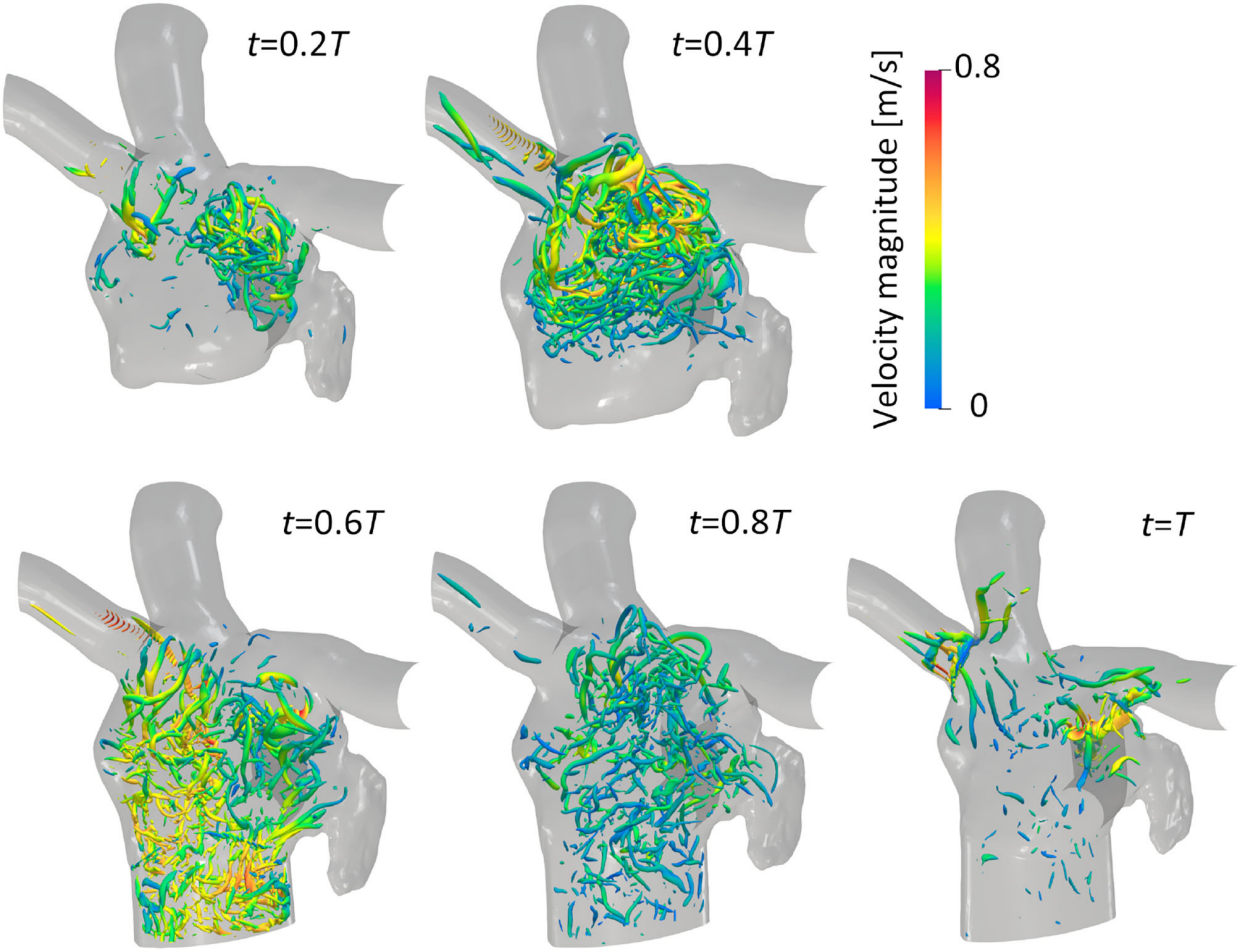
Anterior views of vortex iso-surfaces identified using the λ_2_ criterion (λ_2_ = −0.01 ms^2^) at *t* = 0.2*T*, 0.4*T*, 0.6*T*, 0.8*T*, and *T* before the left upper lobectomy (*T*: cardiac cycle from the start of systole). Contour plots show the velocity magnitude.

**Figure 6:**
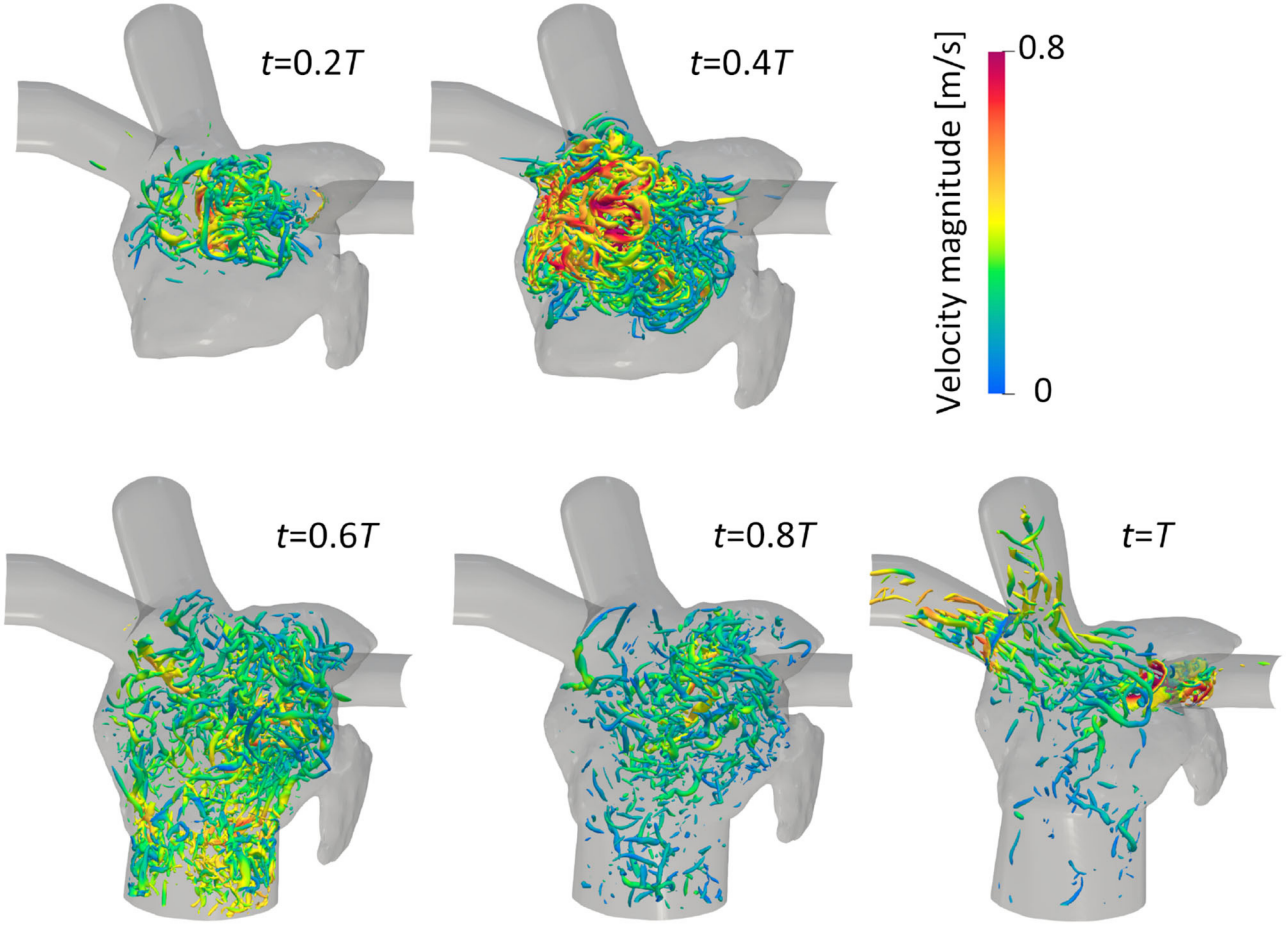
Anterior views of vortex iso-surfaces identified using the λ_2_ criterion (λ_2_ = −0.01 ms^2^) at *t* = 0.2*T*, 0.4*T*, 0.6*T*, 0.8*T*, and *T* after the left upper lobectomy (*T*: cardiac cycle from the start of systole). Contour plots show the velocity magnitude.

The vortex structure and its transition in the LA are next visualized using the λ_2_ criterion (Jeong and Hussain, 1995). Here, λ_2_ is the second-largest eigenvalue of **S**^2^ + **Ω**^2^, where **S** and **Ω** are the symmetric and asymmetric components of ∇**u**, respectively. Figure 5 shows snapshots of the vortex structures in the LA before the LUL; refer to Supplementary Video 2 to see results for a whole cardiac cycle. Before the LUL, vortex rings were found around the PV root and relatively fine-scale vortices appeared in the upper LA region from mid-systole. Although vortex rings around PV roots broke down or became small, fine-scale vortices were transported to the LV at early-diastole, and vortices became sparse at mid-diastole. After the LUL [Figure 6], a relatively dense vortex structure appeared in the upper LA region during systole, and a large portion of these vortices remained even at mid-diastole.

To consider the flow transport profile in the LA and its change due to the LUL, especially in terms of PV inflows, we computed the Lagrange coherent structure (LCS) of the LA blood flow, in which the LCS is locally the most strongly attracting or repelling material surface according to dynamical systems theory (Shadden, 2012; Haller, 2015). Here, we extracted the LCS as iso-surfaces of the finite-time Lyapunov exponent (FTLE) following (Shadden, 2012). The flow map $\phi_{t_{0}}^{t_{0}+\tau }:{\text{x}}_{p}\left(t_{0}\right)\to {\text{x}}_{p}\left(t_{0}+\tau \right)$ was computed with respect to massless tracers **x**_*p*_(*t*_0_) at initial time *t*_0_ under each initial condition by solving the advection in the LA blood flow in finite time τ. The FTLE was then computed at each location as


(5) $$\begin{array}{l} \text{FTLE}\left({\text{x}}_{p},t_{0},\tau \right)=\frac{1}{\left|\tau \right|}\ln{\left\|\frac{\partial \phi_{t_{0}}^{t_{0}+\tau }}{\partial \text{x}}\right\|}_{2}. \end{array}$$

We considered the attracting LCS obtained by integration backward in time (τ < 0), where τ was set at −50 ms following (Seo et al., 2014).

**Figure 7:**
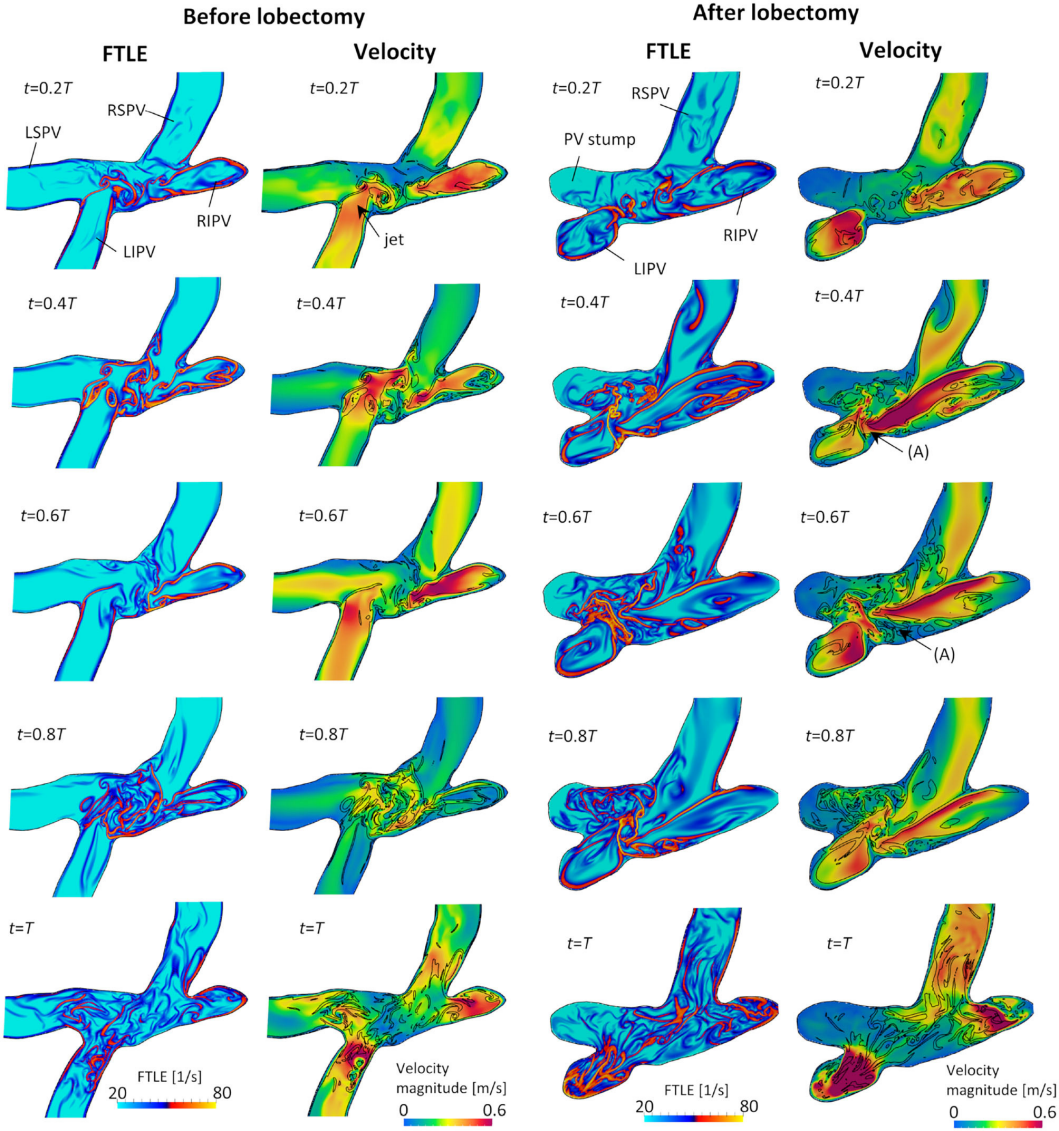
Cross-sectional views of the backward finite-time Lyapunov exponent (FTLE) and velocity distribution with attracting Lagrange coherent structure defined as an iso-surface of an FTLE of 40 s^−1^ at *t* = 0.2*T*, 0.4*T*, 0.6*T*, 0.8*T*, and *T* before and after the left upper lobectomy (*T*: cardiac cycle from start of systole).

Figure 7 shows snapshots of the FTLE and velocity for the LCS extracted from the FTLE on the cross-sectional plane of the LA, including four PVs, before and after the LUL; refer to Supplementary Video 3 to see results for a whole cardiac cycle. Before the LUL, jet flows formed from each PV from mid-systole to early-diastole (*t* = 0.2*T*−0.6*T*) and a clear LCS formed among PV inflows to separate these jets and form swirling flow in the LA. At end-diastole, fine-scale vortices appeared at the center of the LA with decreasing PV inflow, and these vortex structures moved to the PVs and disappeared as the LA contracted (*t* = *T*). In contrast, after the LUL, LCS formed in the direction orthogonal to the flow from left [Figure 7 (arrow (A))] and right PVs at all times during the cardiac cycle except at end-diastole. No swirling flow in the LA was found, and fine-scale vortices appeared at the center of the LA from mid-systole to mid-diastole. LCS formed from the base to the central region of the PV stump but did not form in the deeper region throughout the cardiac cycle.

To understand global energetic states of the LA hemodynamics in a cardiac cycle, we quantified the flow kinetic energy and its viscous dissipation rate, given by


(6) $$\begin{array}{l} \text{Kinetic energy}=\int_{\Omega_{\text{f}}}\frac{1}{2}\rho \left(\text{u}\cdot \text{u}\right)d\Omega , \end{array}$$

(7) $$\begin{array}{l} \text{Dissipation rate}=\int_{\Omega_{\text{f}}}2\eta \text{S}:\text{S}d\Omega . \end{array}$$

**Figure 8:**
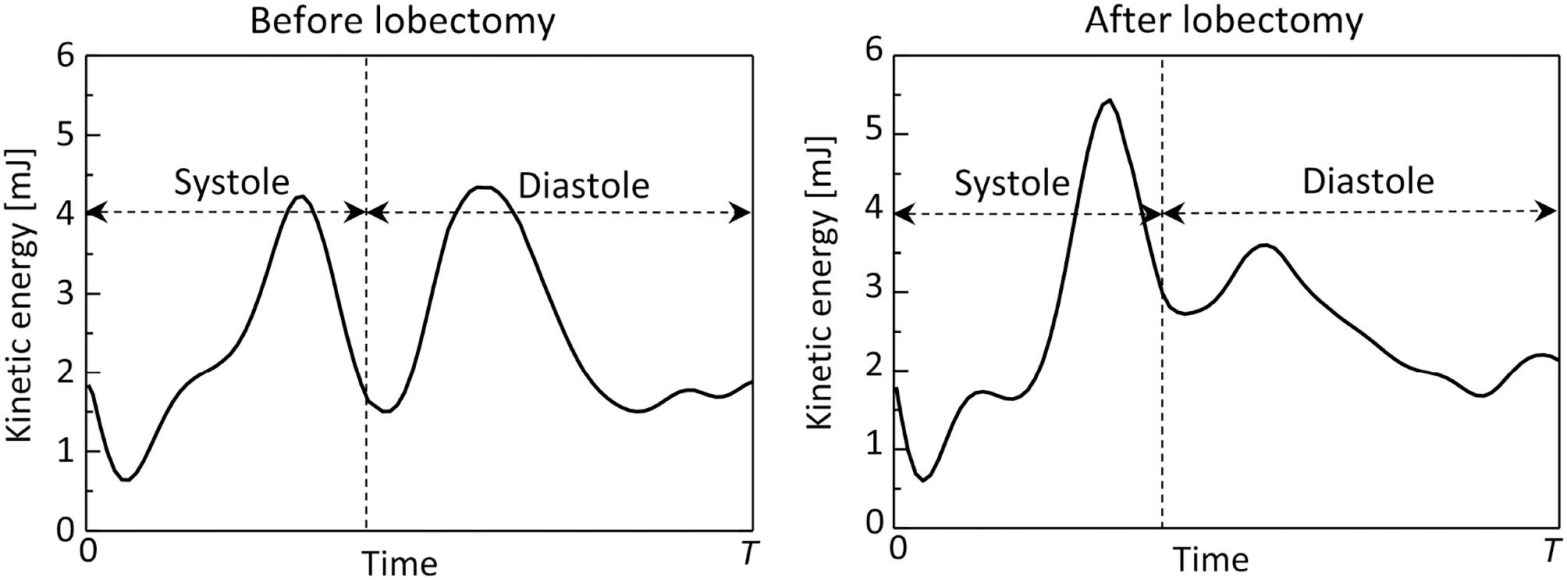
Time courses of the flow kinetic energy in the left atrium throughout the cardiac cycle, before and after the left upper lobectomy (*T*: cardiac cycle from early systole).

**Figure 9:**
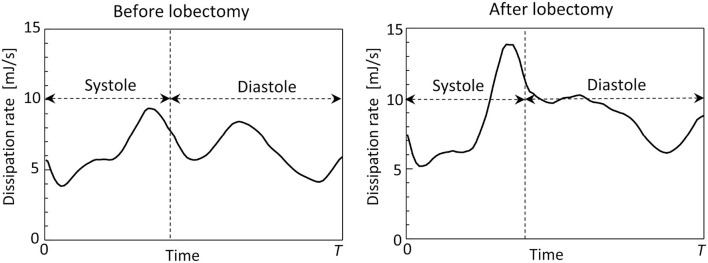
Time courses of the viscous dissipation rate in the left atrium throughout the cardiac cycle, before and after the left upper lobectomy (*T*: cardiac cycle from early-systole).

Figure 8 shows time courses of flow kinetic energy in the LA before and after the LUL. Before the LUL, the flow kinetic energy increased from early-systole to late-systole and temporally decreased at end-systole. The kinetic energy increased again between early-diastole to mid-diastole and decreased until end-systole, then finally increased slightly in response to the atrial kick. After the lobectomy, the first maximum of the kinetic energy at late-systole was relatively high, whereas the second at mid-diastole was relatively low compared with peaks before the LUL. In the time course of the dissipation rate in Figure 9, its value was within 10 mJ/s and moderately increased at late-systole and mid-diastole before the LUL. In contrast, the dissipation rate steeply increased and approached 14 mJ/s at late-systole and remained at approximately 10 mJ/s until mid-diastole after the LUL. These magnitudes after the LUL were constantly higher throughout the cardiac cycle.

## 4. Discussion

The causal relationship between an LUL and LA thrombus formation associated with cerebral infarction has been clarified in several cohort studies conducted over the past 5 years (Yamamoto et al., 2016; Hattori et al., 2019; Riddersholm et al., 2019; Xie et al., 2019), but the underlying mechanism remains an open question. LA hemodynamics, especially in the PV stump, are considered an influential factor leading to thrombus formation (Ohtaka et al., 2014a,b) whereas hemodynamic alteration in the LA after an LUL is poorly understood. The present study is the first to investigate the effects of an LUL on LA hemodynamics using 4D-CT-based computational simulations. The results showed blood flow impingements from the left and right PVs in the LA upper region during almost all cardiac phases after the LUL, and the disappearance of swirling flow observed throughout the LA before the LUL. These results indicate that an LUL affects LA hemodynamics.

Furthermore, to understand transient flow profiles in the LA, we evaluated the LCS of the blood flow, especially the inflow from each PV. The LCS is considered an effective index to intuitively understand cardiovascular hemodynamics with strong unsteadiness (Shadden and Taylor, 2008; Shadden and Arzani, 2015), and this study is the first to quantitative the LCS in the LA and illustrate globally swirling flow before the LUL and flow impingement after the LUL, which are difficult to visualize using conventional approaches such as the λ_2_ criterion. Two main implications of the LCS evaluation are presented below.

Before the LUL, the LCS was found for each PV inflow, especially at mid-systole and early-diastole [Figure 7 (left)]. These results indicate the appearance of jet flows into the LA from each PV and swirling throughout the LA region, as shown in Figure 3 and Supplementary Video 1, which is consistent with known LA flow patterns (Kilner et al., 2000; Fyrenius et al., 2001). After the LUL, LCS formed orthogonal to the PV inflow from the left and right sides and formed fine-scale structures at the center of the LA throughout the cardiac cycle except at end-diastole [Figure 7 (right)]. These characteristic flow patterns indicate PV inflow impingement from the left and right. Moreover, the absence of swirling features in the LA [Figure 3 and Supplementary Video 1] is consistent with the results of a recent MRI study (Nakaza et al., 2021). The flow impingement generated fine-scale vortex structures throughout most of the cardiac cycle [Figure 6] and resulted in relatively high viscous energy dissipation [Figure 9]. The LA blood flow simulations were thus successful in showing possible characteristics of LA hemodynamic alterations due to an LUL.

The LSPV stump due to an LUL is thought to have a relatively high risk of thrombus formation compared with other PV stumps (Ohtaka et al., 2013; Riddersholm et al., 2019; Xie et al., 2019). The LSPV stump is longer than other PV stumps, and several studies have thus hypothesized that blood flow stagnation is enhanced in the LSPV stump (Ohtaka et al., 2013; Xie et al., 2019). Our results show that the flow impingement from PVs generated fine-scale vortex structures in the upper LA region after an LUL [Figure 6], and flow disturbances thus formed around the LSPV stumps. Nevertheless, the LCS formed mainly from the base to the center of the LSPV stump [Figure 7], which indicates that the flow separated between the deep and bottom regions. These results suggest that flow isolation associated with flow stasis is feasible in the deep region of the LSPV stump, whereas flow impingement appears in the LA upper region. This finding supports the current hypothesis that thrombus formation in the LSPV stump is due to flow stasis. The above flow patterns might be affected by boundary conditions in physiological ranges, and further evaluation of the sensitivity of the LCS may therefore give further insights into the underlying mechanism of thrombus formation in the LSPV stump.

This study was the first to investigate LA hemodynamics after an LUL and had several limitations. First, this study demonstrated a possible alteration of LA hemodynamics due to an LUL but did not provide a general description of these effects. Patient-specific differences in anatomical LA geometries and PV stump sizes and connecting angles should be further investigated to determine if the effects observed in this study can be generalized. Although comparisons between clinical cases with and without thrombus formation face difficulties in sample acquisition because thrombus formation is rare (Riddersholm et al., 2019), investigations of LA hemodynamic alterations in additional patient-specific cases may give insight into the possible ranges of flow alteration by an LUL and other PV lobectomies. Second, further investigation of the cause of these characteristic LA hemodynamic alterations is needed. There are three possible causes: an increased remaining PV flow rate due to the LUL, a distribution of flow rates among the PVs, and changes in the relative directions of PVs connecting to the LA. Nakaza et al. (2021) pointed out that the connecting angles of the remaining PVs can be affected by the LUL, especially on the resection side, through remodeling of the remaining lung to expand (Dai et al., 2016). Such alteration of the PV angle on the resection side was observed in this case study (Figure 1). These implications suggest that sensitivities of the PV flow rate and connecting directions to the LA hemodynamics may be attractive indices with which to consider the LA hemodynamic alteration following an LUL.

In addition, the 4D-CT-based computational framework has several limitations. First, time courses of the LV volume extracted from 4D-CT images (Figure 1) have no iso-volumetric contraction and relaxation plateaus, which are difficult to evaluate because of limited temporal resolution. Their absence potentially affects simulated flow characteristics, such as the strength of the reverse flow in the PV. Second, this framework does not consider MV leaflet geometries and the time required for MV opening and closing. These omissions may result in non-smooth changes of the LA flow, making it difficult to quantitate temporal flow alterations during MV opening and closing. Third, this study assumed a uniform PV flow rate based on the work of Lantz et al. (2019), whereas further consideration of patient-specific PV flow distributions can personalize this computational framework. In such cases, further clinical PV flow measurements using echocardiography and cardiac 4D-flow MRI would be helpful. Fourth, vortex visualization indices, such as the λ_2_ criterion, require user-defined thresholds to visualize different vortex scales. For example, simulations of blood flow in the left heart region have focused on relatively large vortex rings around PV roots and its breakdown in the LA (Vedula et al., 2015), while (Mihalef et al., 2011) observed vortex transportation from the LA to LV. Although both vortex characteristics reported above were observed in Figures 5, 6, visualizations of vortex characteristics using different thresholds should be carefully compared.

## 5. Conclusions

This study investigated possible LA hemodynamic alterations caused by an LUL using computational simulations. Computational fluid dynamics simulations of the LA blood flow using a patient-specific LA geometry with motions before and after an LUL were conducted using 4D-CT images. The obtained results demonstrate that the swirling flow pattern throughout the LA region, which was found before the LUL, disappeared after the LUL. Instead, blood flow impingement from left and right PVs appeared in the upper LA region after the LUL and generated fine-scale vortices with relatively high viscous energy dissipation. These findings suggest a strong effect of the LUL on the LA hemodynamics associated with blood flow stasis in the LSPV stump.

## Data Availability Statement

The raw data supporting the conclusions of this article will be made available by the authors, without undue reservation.

## Ethics Statement

The studies involving human participants were reviewed and approved by Institutional Review Board of Jichi Medical University. The patients/participants provided their written informed consent to participate in this study. Written informed consent was obtained from the individual(s) for the publication of any potentially identifiable images or data included in this article.

## Author Contributions

TO implemented the code, performed numerical simulations, and wrote the paper. TY and WY processed the medical images and performed numerical simulations. SE curated the clinical data and reviewed and edited the paper. SW organized the research and reviewed and edited the paper. All authors contributed to the article and approved the submitted version.

## Funding

This work was supported by research grants from JSPS Grants-in-Aid for Scientific Research (18K08796, 19H01175, and 21K18037), Multidisciplinary Research Laboratory System for Future Development, Osaka University (No. j08811), and MEXT as Program for Promoting Researches on the Supercomputer Fugaku (JPMXP1020200401) and used computational resources of supercomputer Fugaku provided by the RIKEN Center for Computational Science (Project ID: hp210181).

## Conflict of Interest

The authors declare that the research was conducted in the absence of any commercial or financial relationships that could be construed as a potential conflict of interest.

## Publisher's Note

All claims expressed in this article are solely those of the authors and do not necessarily represent those of their affiliated organizations, or those of the publisher, the editors and the reviewers. Any product that may be evaluated in this article, or claim that may be made by its manufacturer, is not guaranteed or endorsed by the publisher.

## Figures and Tables

**FIGURE A1:**
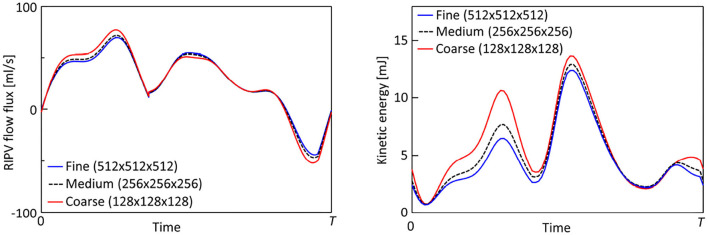
Representative time courses of the flow rate through the right inferior pulmonary vein (left) and flow kinetic energy throughout the computational domain (right) for fine (512 × 512 × 512, grid size = 0.3 mm), medium-resolution (256 × 256 × 256, grid size = 0.6 mm), and coarse (128 × 128 × 128, grid size = 1.2 mm) grids.

**FIGURE A2:**
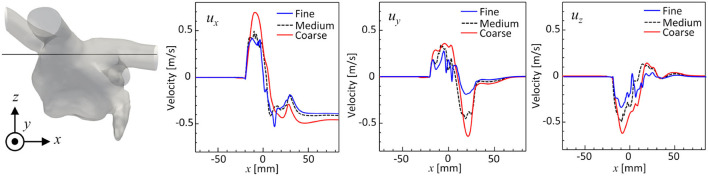
Velocity profiles on the line probe along the x-direction in the upper region of the left atrium at *t* = 0.2*T* for fine (512 × 512 × 512, grid size = 0.3 mm), medium-resolution (256 × 256 × 256, grid size = 0.6 mm), and coarse (128 × 128 × 128, grid size = 0.9 mm) grids.

## Supplementary Material

Supplementary Video 1 Posterior view of the velocity distribution in the left atrium before and after the left upper lobectomy throughout a cardiac cycle.

Supplementary Video 2 Anterior view of the vortex structure transition in the left atrium before and after the left upper lobectomy throughout a cardiac cycle.

Supplementary Video 3 Cross-sectional views of the velocity magnitude, finite-time Lyapunov exponent (FTLE), and Lagrange coherent structure (FTLE iso-surface) in the left atrium before and after the left upper lobectomy throughout a cardiac cycle.

## Appendix. Grid Size Dependencies

A preliminary CFD simulation of blood flow in the LA was conducted on three grids, namely a coarse grid (128 × 128 × 128, resolution of 1.2 mm), medium grid (256 × 256 × 256, resolution of 0.6 mm), and fine grid (512 × 512 × 512, resolution of 0.3 mm) to consider the grid size dependency. Figure A1 shows representative time courses of the flow rate in the right superior pulmonary vein and the flow kinetic energy throughout the computational domain including the LA region and artificially added cylindrical tubes described in section 2.2. The time course of the inflow rate was in excellent agreement at all grid scales. The flow kinetic energy was overestimated using the coarse grid, and values tended to converge as the cell size decreased. Next, to investigate the fine-scale flow profile, we set a line probe in the LA along the *x*-axis of a Cartesian grid [Figure A2 (left)] and evaluated the velocity profile for each coordinate at *t* = 0.2*T*, when the PV inflow velocity was high [Figure A2 (right)]. In all cases, small flow disturbances were found using the fine grid owing to LA flows in the transition regime between laminar and turbulent flows. These preliminary findings showed that the variability of the results decreased commensurately with the grid size, and differences between the medium and fine grids were sufficiently small compared with those observed before and after the LUL (Section 3). We, therefore, concluded that the fine grid was suitable for the computations in this study.
